# Focal Adhesion Kinase contributes to insulin-induced actin reorganization into a mesh harboring Glucose transporter-4 in insulin resistant skeletal muscle cells

**DOI:** 10.1186/1471-2121-9-48

**Published:** 2008-09-04

**Authors:** Bharti Bisht, Chinmoy S Dey

**Affiliations:** 1 Signal Transduction Research Laboratory, Department of Biotechnology, National Institute of Pharmaceutical Education and Research (NIPER), Sector 67, SAS Nagar, Punjab, 160 062, India; 2 Current address : Department of Biology, Indian Institute of Science Education and Research (IISER) Mohali, MGSIPA Complex, Sector 26, Chandigarh, 160019, India

## Abstract

**Background:**

Focal Adhesion Kinase (FAK) is recently reported to regulate insulin resistance by regulating glucose uptake in C2C12 skeletal muscle cells. However, the underlying mechanism for FAK-mediated glucose transporter-4 translocation (Glut-4), responsible for glucose uptake, remains unknown. Recently actin remodeling was reported to be essential for Glut-4 translocation. Therefore, we investigated whether FAK contributes to insulin-induced actin remodeling and harbor Glut-4 for glucose transport and whether downregulation of FAK affects the remodeling and causes insulin resistance.

**Results:**

To address the issue we employed two approaches: gain of function by overexpressing FAK and loss of function by siRNA-mediated silencing of FAK. We observed that overexpression of FAK induces actin remodeling in skeletal muscle cells in presence of insulin. Concomitant to this Glut-4 molecules were also observed to be present in the vicinity of remodeled actin, as indicated by the colocalization studies. FAK-mediated actin remodeling resulted into subsequent glucose uptake via PI3K-dependent pathway. On the other hand FAK silencing reduced actin remodeling affecting Glut-4 translocation resulting into insulin resistance.

**Conclusion:**

The data confirms that FAK regulates glucose uptake through actin reorganization in skeletal muscle. FAK overexpression supports actin remodeling and subsequent glucose uptake in a PI3K dependent manner. Inhibition of FAK prevents insulin-stimulated remodeling of actin filaments resulting into decreased Glut-4 translocation and glucose uptake generating insulin resistance. To our knowledge this is the first study relating FAK, actin remodeling, Glut-4 translocation and glucose uptake and their interrelationship in generating insulin resistance.

## Background

The uptake of glucose by skeletal muscle, a major insulin responsive organ, is mediated by the insulin-responsive glucose transporters, Glut-4 [1,2]. Insulin stimulation elicits Glut-4 recruitment to the plasma membrane of skeletal muscle thereby increasing glucose influx [3,4]. Under insulin resistant condition, loss of this response triggers an elevation of circulating insulin, resulting into hyperinsulinemia. This worsens insulin resistance resulting into type 2 diabetes [5-7]. Therefore, an in-depth study of insulin signaling at the cellular and molecular level is critical to understand the pathogenesis of type 2 diabetes.

In order to better understand insulin resistance at a molecular level we have previously developed an insulin resistant skeletal muscle cell model by differentiating C2C12 skeletal muscle cells in chronic presence (MFI) and absence (MF) of insulin in serum free medium [8]. The model mimics the physiological insulin resistant condition of skeletal muscle. Using the model system we have recently reported involvement of Focal Adhesion Kinase (FAK), a cytosolic non-receptor protein tyrosine kinase, in the regulation of insulin resistance in C2C12 skeletal muscle by modulating glucose uptake [9]. The study established that under insulin resistant condition overexpression of FAK enhanced insulin-mediated glucose uptake. However, no change in the expression of Glut-4 was observed due to overexpression of FAK, thereby leaving an important unresolved issue of how FAK regulates glucose uptake under insulin resistant condition.

Recent reports suggest that insulin induces remodeling of actin filaments, in myotubes [10,11], adipocytes [12,13] and fibroblasts [14] under normal physiological conditions. Moreover, disruption of actin cytoskeleton was also reported to prevent translocation of Glut-4 to the cell surface of muscle [10,15-17]. In fibroblasts FAK was reported to stabilize microtubules and its tyrosine kinase activity was reported to play an important role in the rearrangement of actin fibers in CHO/IR cells [18-20]. Recently it was reported that *Fak*-null keratinocytes display an aberrant actin cytoskeleton, which is tightly associated with focal adhesions and microtubules [21].

Therefore, in the light of the above literature, we sought out to determine whether FAK regulates glucose uptake by altering actin filament dynamics and Glut-4 translocation, in insulin responsive and resistant skeletal muscle cells.

## Results

### Insulin-mediated colocalization of FAK with remodeled actin

**Figure 1:**
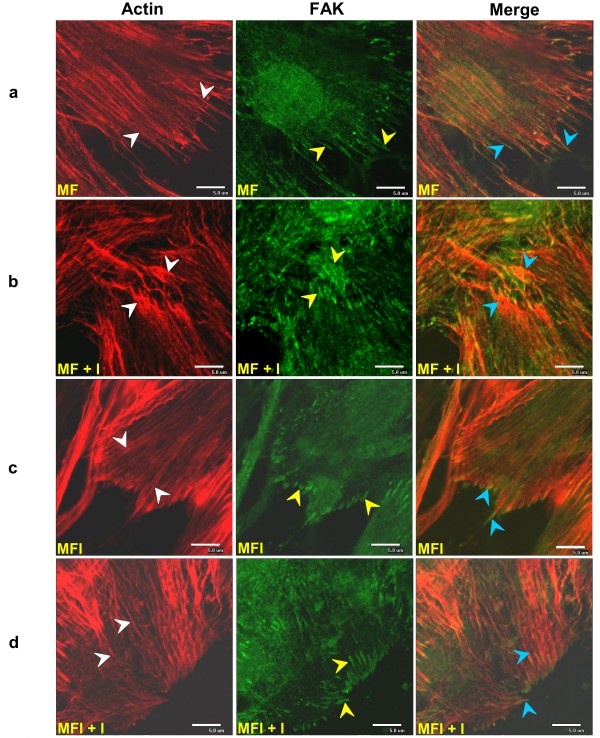
**Effect of insulin on subcellular distribution of FAK and Actin**. C2C12 cells were differentiated under insulin sensitive (MF) and insulin resistant (MFI) condition and stimulated with or without insulin for 30 min at 37°C after 4 h starvation followed by fixation and permiabilization and probed with anti-FAK antibody. Cells were subjected to immunofluorescence microscopy by using anti-mouse FITC-conjugated secondary antibody for FAK and Phalloidin Texas Red for actin. Bar corresponds to 5 μm. Images were captured from different fields and a representative image of 3 experiments is presented. a: C2C12 cells differentiated under MF condition, b: C2C12 cells differentiated under MF condition, stimulated with insulin, c: C2C12 cells differentiated under MFI condition, d: C2C12 cells differentiated under MFI condition, stimulated with insulin. White arrow head represent actin filaments, Yellow arrow head represent FAK localization and Blue arrow head indicates colocalized FAK and actin.

**Figure 2:**
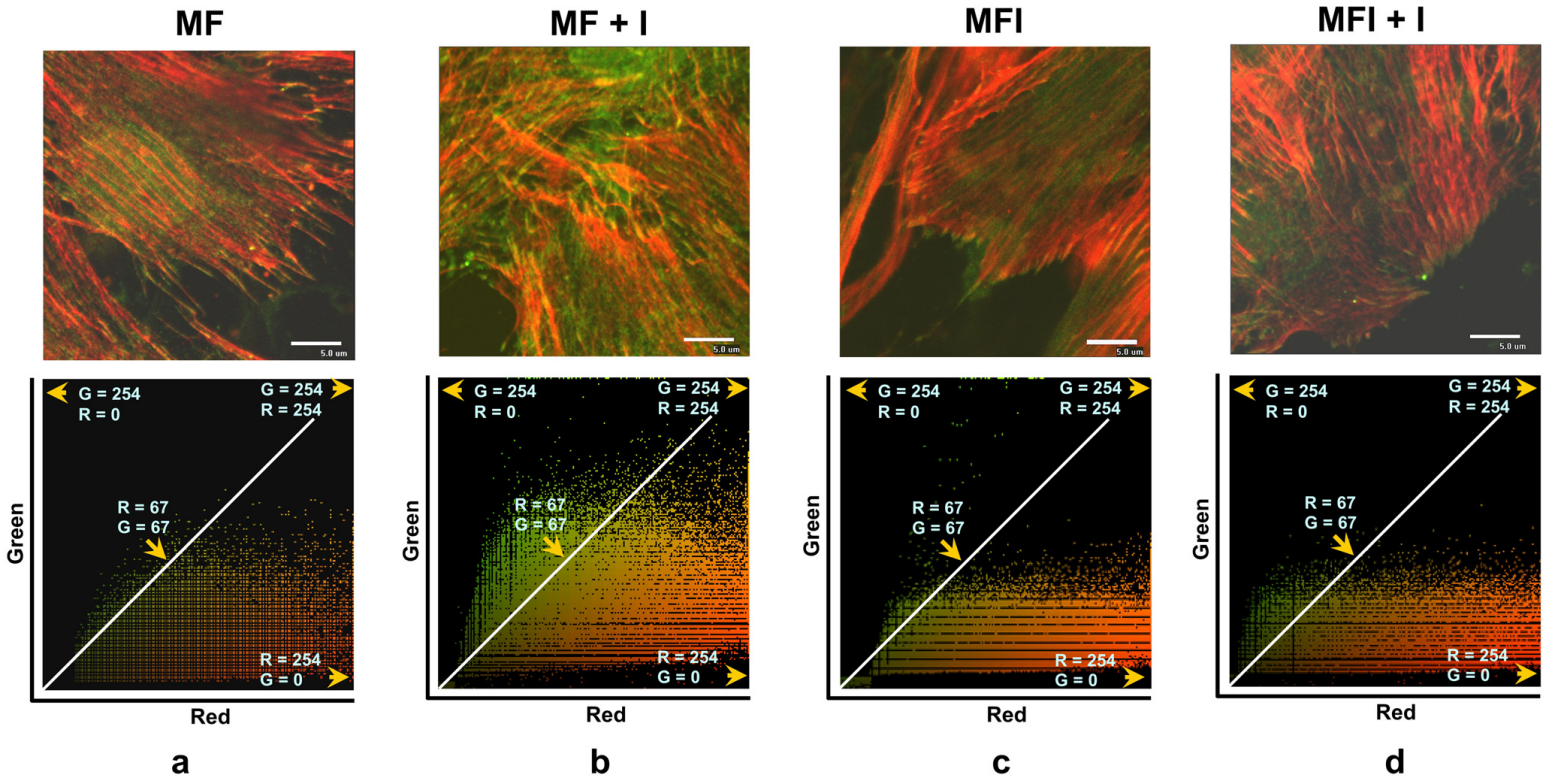
**Effect of insulin on FAK and Actin colocalization**. Colocalization graphs of a, b, c and d of Figure 1 for FAK and actin. Colocalization graph represents analysis of the localized pattern of green and red in C2C12 cells. Colocalization graph (Scatter Plot) shows color intensity distribution of pixels. X axis represent red color channel and Y axis represent green color channel. The white line running diagonally demonstrates colocalized pixels.

In order to investigate the role of FAK in regulating insulin-induced actin remodeling and subsequent Glut-4 translocation, if any, we examined the effect of insulin on the distribution and localization of FAK and actin cytoskeleton rearrangements in insulin sensitive and resistant skeletal muscle cells. C2C12 skeletal muscle cells were differentiated under insulin sensitive (MF) and insulin resistant (MFI) conditions, stimulated with or without insulin (30 min with 100 nM insulin), immunostained with anti-FAK antibody and phalloidin Texas Red and were observed under a confocal microscope (Fig. 1). Immunocytochemical analysis revealed that under basal condition (in absence of insulin stimulation) FAK colocalized with actin filaments only at the site of adhesion of insulin sensitive skeletal muscle cells (MF) (Fig. 1, panel a). However insulin stimulation caused remodeling of actin filaments into a cortical mesh (Fig. 1, panel b, red) and FAK was found to be redistributed and colocalized with remodeled actin filaments, (Fig. 1, panel b) as compared to unstimulated cells. The observed phenomenon of cellular colocalization between FAK and actin was elucidated by colocalization graph (two color scatter plot) plotted between the green and red color intensity pixels by EZ Viewer software (Fig. 2). Colocalization graph under MF condition showed no colocalization between FAK and actin at the cortex as indicated by the pixels present distributed along x and y axis (Fig. 2, panel a). However, significant colocalization was observed in the cortical section after insulin stimulation (Fig. 2, panel b).

On the other hand, in insulin resistant skeletal muscle (MFI), neither actin remodeling nor redistribution of FAK was observed with or without insulin stimulation (Fig. 1, panel c, d). No colocalization was observed between FAK and actin at the cortical sites (Fig. 2, panel c, d). Taken together, these results indicate that in insulin sensitive skeletal muscle cells insulin stimulation causes redistribution of FAK leading to its colocalization with remodeled actin filaments. However, no such event takes place in insulin resistant cells.

### Effect of modulation of FAK expression on actin remodeling and Glut-4 translocation

Previously we have reported that overexpression of FAK increases insulin-stimulated glucose uptake without altering Glut-4 expression [9], thereby, pointing to a possibility of enhanced Glut-4 translocation. In order to investigate the significance of FAK and actin colocalization on subsequent translocation of Glut-4 in skeletal muscle, we had generated a C2C12 skeletal muscle stable cell line overexpressing Green Florescence Protein (GFP) tagged Glut-4 molecules (C2Glut-4^wt/+^, see Additional file 1). In order to establish that overexpressed GFP-Glut-4 cycles and respond to insulin in a manner similar to endogenous Glut-4, transfected cells were differentiated and stimulated with or without insulin and Glut-4 translocation was examined by confocal microscopy (see Additional file 2). Positive response of the transporters to insulin stimuli suggested the existence of functional Glut-4 translocation machinery. Our observation is in coherence with the previous report suggesting that C2C12 myotubes were equipped with the basic Glut-4 translocation machinery and could be activated by insulin [22]. Thus proposes the above cell line as a useful model for analyzing Glut-4 translocation in skeletal muscle.

To further investigate the participation of FAK in actin remodeling, two approaches were used: gain of function by increasing the expression of FAK and loss of function by specifically knocking down the expression of FAK. For overexpression C2Glut-4^wt/+ ^cells were transfected with a plasmid containing the wild type FAK gene (C2Glut-4^wt/+^FAK^wt/+^) and knocked down by using a FAK specific siRNA (C2Glut-4^wt/+^FAK^wt/-^). The FAK specific siRNA sequences were designed by Quiagen GmbH (Germany) and have been utilized previously in our laboratory to specifically inhibit FAK expression in C2C12 skeletal muscle cells [9]. To determine the extent of overexpression, cells were differentiated, lysed and subjected to westernimmunoblotting, probed with anti-FAK antibody (see Additional file 3) and compared with the untransfected control. The significant amount of overexpression (more than 70%, see Additional file 3, lane 3 vs lane 1) of FAK was observed. An additional data on silencing of FAK by siRNA (more than 90%, see Additional file 3, lane 4 vs lane 1) has also been presented. Thus, various cell models (over and under expressing FAK and over expressing Glut-4) were generated and validated, for investigating FAK-mediated effect of insulin on actin remodeling and Glut-4 translocation.

**Figure 3:**
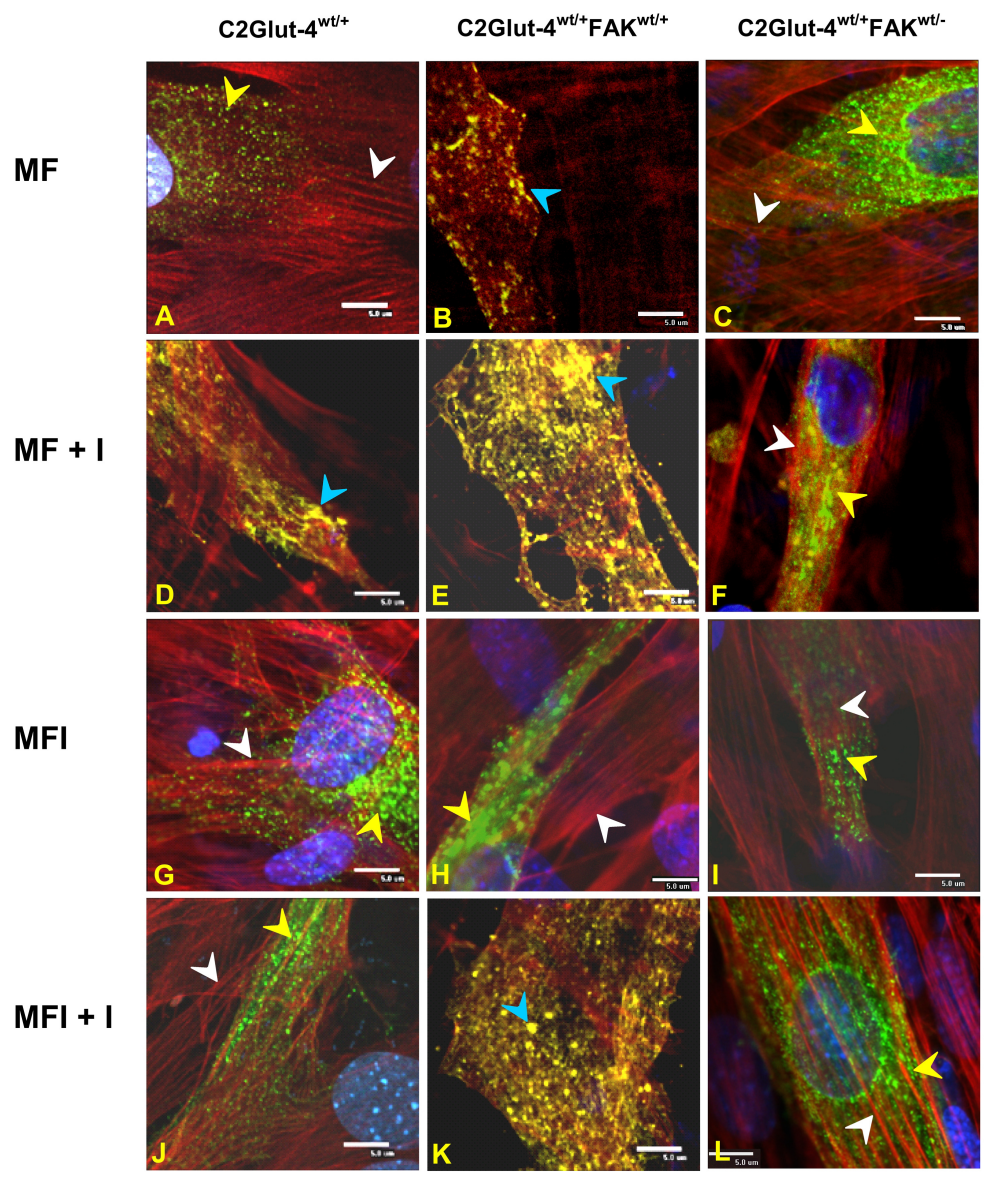
**Effect of FAK expression modulation on insulin-mediated actin remodeling and Glut-4 translocation**. Glut-4 transfected cells expressing endogenous level of FAK (C2Glut-4^wt/+^), overexpressing FAK (C2Glut-4^wt/+^FAK^wt/+^) and underexpressing (silenced) FAK (C2Glut-4^wt/+^FAK^wt/-^) were differentiated under MF and MFI condition, serum starved (4 h) and stimulated with and without 100 nM insulin for 30 min at 37°C followed by fixation and permeabilization. Actin filaments were labeled with Phalloidin Texas Red, nucleus with DAPI. Images were captured from different fields and a representative image of 3 experiments is presented. Bar represents 5 μm. A, B and C: C2Glut-4^wt/+^, C2Glut-4^wt/+^FAK^wt/+ ^and C2Glut-4^wt/+^FAK^wt/- ^cells differentiated under MF condition; D, E and F: C2Glut-4^wt/+^, C2Glut-4^wt/+^FAK^wt/+ ^and C2Glut-4^wt/+^FAK^wt/- ^cells differentiated under MF condition stimulated with insulin (MF+I); G, H and I: C2Glut-4^wt/+^, C2Glut-4^wt/+^FAK^wt/+ ^and C2Glut-4^wt/+^FAK^wt/- ^cells differentiated under MFI condition; J, K and L: C2Glut-4^wt/+^, C2Glut-4^wt/+^FAK^wt/+ ^and C2Glut-4^wt/+^FAK^wt/- ^cells differentiated under MFI condition stimulated with insulin (MFI+I). White arrow head represent actin filaments, Yellow arrow head represent Glut-4 molecules and Blue arrow head indicates colocalized actin with Glut-4.

FAK untransfected (C2Glut-4^wt/+^) and transfected (C2Glut-4^wt/+^FAK^wt/+ ^and C2Glut-4^wt/+^FAK^wt/-^) cells were differentiated under MF and MFI conditions and stimulated with and without insulin and subjected to immunoflorescence microscopy. In absence of insulin stimulation, MF cells expressing end ogenous level of FAK showed long actin filaments and no colocalization between actin and Glut-4 at the cortical region (Fig. 3, panel A). (For easier comparison only merge images are shown. For complete panel (GFP, Phalloidin, DAPI and Merge) see Additional file 4, panel A). No significant change in the structure of actin filaments and Glut-4 molecules were observed in cells with underexpressed FAK (Fig. 3, panel C and see Additional file 6, panel A). A small amount of actin remodeling was observed with cells overexpressing FAK (Fig. 3, panel B) but this was not significant as compared to what was observed after insulin stimulation. Whereas after insulin stimulation, actin was found to be reorganized at the perinuclear region upward into a mesh-like structure showing colocalized Glut-4 molecules (Fig. 3, panel D and see Additional file 4, panel B). Interestingly, the extent of actin remodeling after insulin stimulation was found to be significantly increased in FAK overexpressed cells (Fig. 3, panel E and see Additional file 5, panel B) as compared to C2Glut-4^wt/+ ^cells (Fig. 3, panel E vs. Fig 3, panel D). In presence of insulin, overexpression of FAK reorganized filamentous actin into a mesh-like structure (see Additional file 5, panel B). In addition, a huge amount of colocalized Glut-4 molecules were also observed in the same section of the remodeled actin. Data suggest that overexpression of FAK enhances insulin-mediated actin remodeling and remodeled actin harbors the Glut-4 molecules which eventually facilitate the translocation of these transporters to the plasma membrane. Consistent to the previous report of Li *et al*., [23], we did not observe a clear shift of Glut-4 molecule to perinuclear region forming a tighter ring as observed in adipocytes after insulin stimulation [24,25]. In contrast to the C2Glut-4^wt/+ ^cells, FAK silenced cells did not show actin remodeling (Fig. 3, panel F) thus, further confirming the role of FAK in governing actin dynamics.

Under insulin resistant condition (MFI) no actin remodeling was observed in C2Glut-4^wt/+ ^cells (Fig. 3, panel G, see Additional file 4, panel C). C2Glut-4^wt/+^FAK^wt/+ ^and C2Glut-4^wt/+^FAK^wt/- ^cells did not show any significant change in actin dynamics (Fig. 3, panel H and I). Even after insulin stimulation C2Glut-4^wt/+ ^cells under MFI condition did not show any alteration of actin dynamics (Fig. 3, panel J). However, overexpression of FAK under MFI condition augmented actin remodeling after insulin stimulation (Fig. 3, panel K) but lesser than that observed under MF condition (Fig. 3, panel K vs. Fig. 3, panel E). These results are consistent with our previous reports that, insulin stimulation causes enhanced glucose uptake under MF as well as in MFI condition in FAK overexpressing cells [9]. The data suggests that overexpression of FAK under insulin resistant condition alters insulin-mediated actin remodeling and thereby regulates glucose uptake by differentially harbouring Glut-4 molecules. Under FAK silenced condition (C2Glut-4^wt/+^FAK^wt/-^cells), the insulin-mediated additional Glut-4 redistribution, actin remodeling, and colocalization were largely abolished in MFI condition (Fig. 3, panel L). The data thus strongly suggests that FAK regulate insulin-mediated actin remodeling into a mesh harboring Glut-4 molecules.

### Role of Phosphatidylinositol-3 Kinase (PI3K) in FAK-mediated regulation of actin remodeling

**Figure 4:**
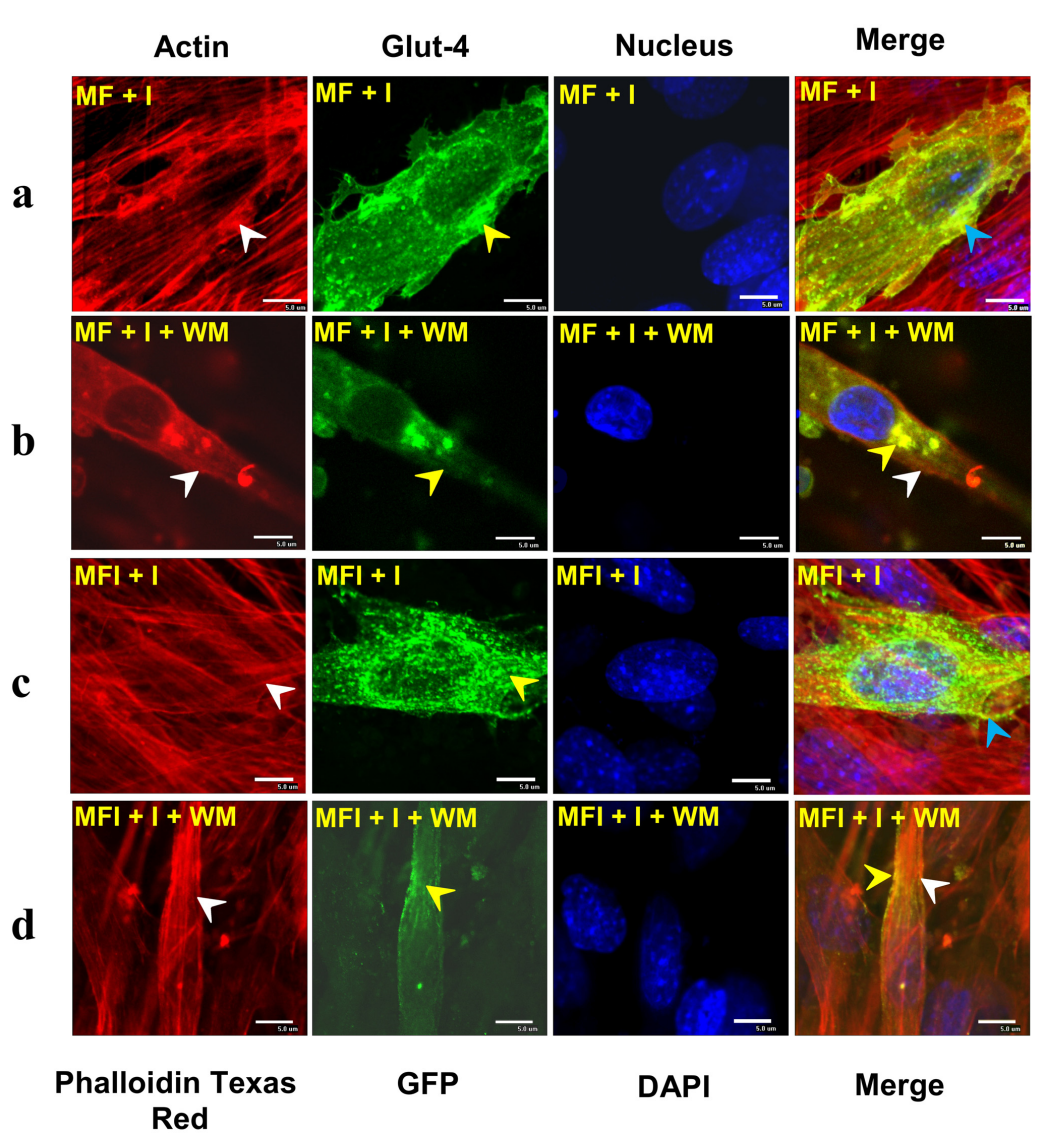
**Effect of wortmannin (WM) on insulin-mediated colocalization of remodeled actin, Glut-4 molecule in C2Glut-4^wt/+^FAK^wt/+ ^cells**. Serum starved (4 h) C2Glut-4^wt/+^FAK^wt/+ ^myotubes were differentiated under MF and MFI condition and stimulated with insulin (100 nM) for 30 min. Cells were preincubated with 200 nM WM for 30 min, followed by insulin-stimulation (100 nM) for 30 min under MF and MFI condition. Cells were fixed, permeabilized, stained with Phalloidin Texas Red and nucleus was stained with DAPI. Images were captured from different fields and a representative image of 3 experiments is presented. Bar represents 5 μm. a: Cells differentiated under MF condition stimulated with insulin; b: Cells differentiated under MF condition, treated with WM and stimulated with insulin; c: Cells differentiated under MFI condition stimulated with insulin; d: Cells differentiated under MFI condition, treated with WM and stimulated with insulin. White arrow head represent actin filaments, Yellow arrow head represent Glut-4 molecules and Blue arrow head indicates colocalized actin with Glut-4.

**Figure 5:**
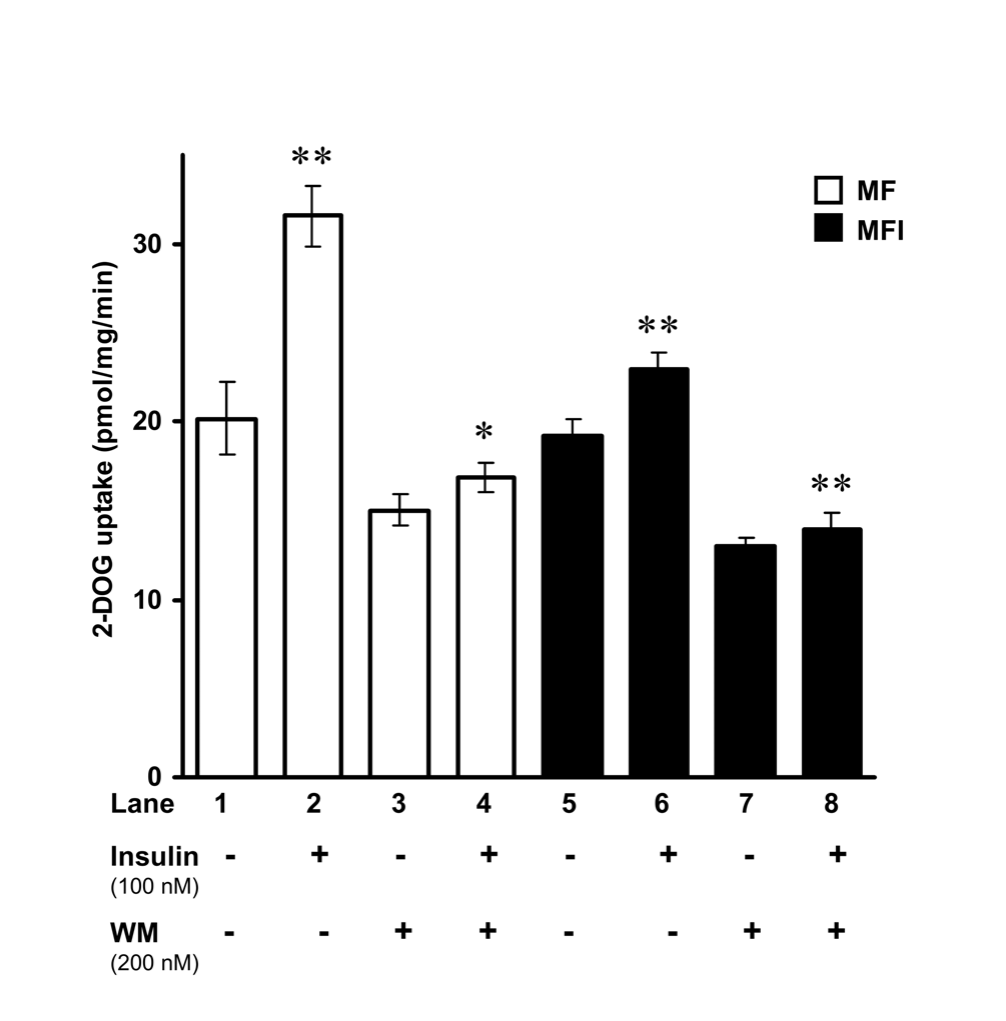
**Effect of wortmannin (WM) on insulin-mediated glucose uptake in C2Glut-4^wt/+^FAK^wt/+ ^cells**. C2Glut-4^wt/+^FAK^wt/+ ^cells were differentiated under MF and MFI condition, serum starved for 1 h and were stimulated with insulin (100 nM) for 15 min, preincubated with 200 nM WM for 30 min, followed by 2DOG uptake in 40 μg cell lysates. The uptake was measured in duplicates. Data has been presented in terms of pmol/mg/min. Open bars represent cells under MF condition, Closed bars represents cells under MFI condition. Experiments were repeated thrice. Values are mean ± SEM. *P < 0.05, **P < 0.01 compared to lane 1.

Earlier we had reported that FAK silencing develops insulin resistance via inactivation of PI3K activity [9]. Altered expression of FAK was observed to modulate actin dynamics, hence, we examined whether FAK regulates actin remodeling and subsequent Glut-4 translocation in a PI3K-dependent pathway. To determine this, the downstream pathway was disrupted by inhibiting PI3K activity using wartmanin (WM). C2Glut-4FAKwt/^+ ^cells were treated with wartmanin (WM), stimulated with insulin, stained for actin and subjected to fluorescence microscopy. We observed significant amount of remodeling in C2Glut-4FAKwt/^+ ^cells after insulin stimulation under both MF and MFI conditions (Fig. 4, panel a, c), whereas WM treatment abolished the observed effect (Fig. 4, panel b, d). To further substantiate the effect of WM, glucose uptake of C2Glut-4FAKwt/^+ ^cells was assayed under MF and MFI condition. As a result of WM treatment a significant decrease (53.3 ± 0.34%) in insulin-stimulated glucose uptake (Fig. 5, lane 3 vs. 2; *P *< 0.01) was observed and was not restored even after insulin stimulation (Fig. 5, lane 4 vs. 2; *P *< 0.01) under MF and MFI conditions. Therefore, FAK regulates insulin-induced actin remodeling harboring Glut-4 in a PI3K-dependent pathway in insulin responsive and resistant cell lines. Data provides mechanistic insight of FAK-mediated regulation of insulin resistance.

## Discussion

Huang *et al*., [26] has recently reported that reducing FAK expression results into "fewer stress fibers" and "scarce focal adhesion complexes" under normal insulin signaling and proposed a probable theoretical signaling cascade linking FAK, actin and Glut-4. However, the interrelationship between FAK, actin dynamics and Glut-4 translocation remains unaddressed as is the direct proof on FAK's possible role in cascading actin dynamics and Glut-4 translocation in regulating insulin resistance. It is well recognised that insulin causes a rapid and marked "remodeling" of actin filaments, in different cells like myotubes [10], adipocytes [12,27,28] and fibroblasts [15,18] for the translocation of Glut-4 molecules. Therefore, it is not the number of stress fibers, as observed by Huang *et al*., [26] it is the actin remodeling which is considered as the biophysical interacting module by which the probable interaction between actin and Glut-4 translocation is brought into reality. Regulation of glucose uptake in muscle cells via Glut-4 is a fundamental action of insulin and gets disrupted in insulin resistance. As the disease is expanding in epidemic proportions, understanding the molecular basis of Glut-4 translocation especially under the insulin resistant condition is paramount. Hence, the present study was designed to explore the fundamentally unresolved issue of how FAK regulates Glut-4 translocation and thereby establishes the biological function of FAK in both insulin responsive and resistant cell lines. This has been explored by overexpression of FAK and perturbing endogenous FAK expression. We observed the following: (i) FAK regulates insulin-stimulated "actin remodeling" leading to Glut-4 translocation (ii) inhibition of FAK prevents actin remodeling and decreases glucose uptake, (iii) under insulin resistant condition, overexpression of FAK induces insulin-stimulated actin remodeling, colocalization with Glut-4 prior to translocation and (iv) PI3K is upstream to actin colocalization. Therefore we are reporting for the first time, a pathophysiological role of FAK-mediated actin remodeling induced Glut-4 translocation and glucose uptake in both insulin responsive and resistant cell lines.

Several laboratories have suggest that cytoskeletal mechanics plays an essential role in insulin-dependent Glut-4 translocation [29]. Studies examining the role of actin cytoskeleton in this process in L6 myotubes and adipocytes have shown that disruption of actin cytoskeleton inhibits insulin-mediated Glut-4 translocation [10,28]. Recently, cytoskeletal abnormalities were reported in insulin resistant skeletal muscle cells [30]. Though, growth factor-induced role of FAK with actin was also reported where increased FAK tyrosine phosphorylation was observed to enhance the formation of actin stress fibers in Chinese Hamster Ovary cells overexpressing the human insulin receptor (CHO/IR). But no physiological role of FAK tyrosine phosphorylation linking actin reorganization was reported [18]. FAK was also reported to regulate cellular morphology via actin cytoskeleton in acinar cells [31]. FAK prompted p190Rho-GEF tyrosine phosphorylation after integrin or growth factor stimulation in neuro2A cells, which activates actin stress fiber formation leading to morphological changes in actin cytoskeleton [32]. However, to the best of our knowledge, no study was available establishing the role of FAK in altering actin remodeling and subsequent Glut-4 translocation in insulin resistant condition.

Previous studies indicate that insulin stimulation rearranges filamentous actin into mesh-like structures at the cortex [23]. However, the nature and characteristics of actin filament remodeling has not been elucidated earlier. In the present study we observed that although FAK normally localized at the focal adhesions and colocalized with actin at the growing ends of cells. However, after insulin stimulation FAK was redistributed and were found to be colocalized at the site of remodeled actin. Colocalization of FAK with remodeled actin shows that remodeled filaments with actively growing ends govern rapid polymerization/depolymerization as molecular motors which function as a conveyor belt translocating Glut-4. It has been previously reported that insulin facilitates the association of PI3K with Glut-4 vesicles by inducing actin remodeling and recruits Glut-4 to the plasma membrane [33]. Our observation indicates that after insulin stimulation FAK localizes to the actin remodeling sites which acts as a hub providing a platform for insulin signalling proteins (e.g. PI3K etc) for interaction. This in turn facilitates Glut-4 translocation. Our study thus provides a conceptual advancement regarding the cellular localization and distribution of FAK with the remodeled actin filaments and its connectivity with glucose uptake.

Previous reports suggest that insulin modulates actin dynamics in PI3K-dependent and -independent ways [34-37]. We have previously reported that FAK regulates insulin signaling by altering the activity of PI3K [9]. Downstream to PI3K a divergence of signal takes place where signal passes primarily through PKC [9]. Our present study demonstrates that FAK regulates Glut-4 translocation through PI3K. A recent study of Liu *et al*., [38] has reported involvement of PKC in insulin-induced glucose transport through actin remodeling. Therefore, considering all these studies together we propose that FAK regulates insulin signaling through activation of IRS-1, PI3K, Akt, PKC, actin remodeling, Glut-4 translocation and glucose uptake (see Additional file 7).

## Conclusion

Hence, the present study provides insights to the molecular mechanism of FAK-mediated Glut-4 translocation and glucose uptake via actin remodeling and its correlation with the pathophysiology of insulin resistance in skeletal muscle, a tissue responsible for 80% of postprandial glucose disposal and a major peripheral site of insulin resistance in diabetes. Extrapolating the finding of the study we can conclude that overexpression/activation of FAK under resistance condition can be a potential therapeutic approach towards insulin resistance.

## Methods

### Materials

DMEM and trypsin-EDTA were purchased from Gibco BRL (Grand Island, NY). FCS was from Biological Industries (Kibbutz Beit, Haemek, Israel). Nutrient Mixture F-12 Ham, MCDB 201 medium, BSA (cell culture grade), [^3^H]2-deoxyglucose (2DOG), TEMED, anti-rabbit, anti-mouse IgG alkaline phosphatase and FITC conjugated were from Sigma Chemical Company (MO, USA). Anti-FAK antibody was from Santa Cruz Biotechnology (CA, USA). Bovine insulin, wartmanin and anti-Glut-4 antibody was purchased from Calbiochem (CA, USA). 4,6-diamidino-2-phenylindole (DAPI), Phalloidin Texas Red were purchased from Molecular Probes, Inc. TransFast transfection reagent was from Promega (Madison, WI). RNAifect transfecton reagent was from Qiagen GmbH (Germany). All the other reagents unless attributed specifically were from Sigma Chemical Company.

### Cell culture

C2C12 skeletal muscle (wild-type and transfectants) were maintained in DMEM supplemented with 15% FCS (containing penicillin 100 IU/ml, streptomycin 100 μg/ml) in 5% CO_2 _at 37°C. 70% confluent cells were differentiated in an equal mixture of MCDB 201 and Ham's F-12 medium in absence (MF: insulin sensitive) or in chronic presence of 100 nM insulin (MFI: insulin resistant) for 3 days [8].

### Transfection of plasmid vectors

C2C12 cells were transfected with pcDNA3-GFP-Glut-4 plasmid and pcDNA3.1-HA-FAKwt plasmid using TransFast transfection reagent as describe earlier [39].

### siRNA transfection

siRNA oligonucleotides against FAK targeted sequence 5'-TGCAATGGAACGAGTATTAAA-3', designed and synthesized by Qiagen GmbH (Germany) were transfected (400 nM) in C2C12 cells using RNAifect transfection reagent in reduced serum Opti-MEM as describe earlier [9].

### Cell lysis and westernimmunoblotting

Cells were washed with ice cold PBS and lysed in lysis buffer and equal concentration of lysates was subjected to SDS-PAGE followed by westernimmunoblotting as reported before [9].

### Fluorescence and Confocal Laser Microscopy

Immunofluorescence studies of C2C12 cells (transfected and untransfected) were carried out as described previously [11,38]. Briefly, myotubes were subjected to starvation for 4 h and treated with 100 nM insulin for 30 min at 37°C. Cells were fixed with 2% paraformaldehyde in 0.1 M sodium phosphate buffer, pH 7.3 for 30 min. Cells were washed in PBS containing 1% BSA and blocked using blocking buffer (BSA 1%, goat serum 2% in PBS) for 30 min. Cells were permeabilized by incubating with 0.2% Triton X-100 for 10 min, followed by washing with PBS/BSA solution. Cells were incubated with anti-FAK antibody for 2 h at room temperature. Bound antibody was visualized under microscope (Nikon, Tokyo, Japan) by incubating with secondary antibody labeled with FITC. For labeling of actin filaments, fixed and permeabilized cells were incubated for 1 h at room temperature with Phalloidin Texas Red (0.01 U/coverslip). To assess autofluorescence, additional samples were treated for 1 h with PBS without labeled phalloidin. For nuclear staining, cells were labeled with 600 nM DAPI in PBS for 5 min at room temperature. Cells were washed further with PBS and mounted in mowiol on to glass slides. Digital micrographs were taken using a Nikon camera mounted on Nikon E600 microscope using 60 × confocal objective. Images were processed using Image-Pro Express (Media Cybernetics, Madison, U.S.A.), EZ Viewer and Adobe Photoshop 5.5 (Adobe Systems Inc., CA, U.S.A.) software. At least 10 microscopic fields were observed for each sample.

### Glucose uptake assay

Glucose uptake assays were performed using 2-DOG as earlier described [9].

### Statistical analysis

Data are expressed as mean ± SEM. For comparison of two groups, *P*-values were calculated by two-tailed unpaired student's t-test. In all cases *P *< 0.05 was considered to be statistically significant.

## Abbreviations

FAK: focal adhesion kinase. PI3K: phosphatidylinositol 3'-kinase. GFP: green fluorescent protein. DAPI: 4,6-diamidino-2-phenylindole. 2DOG: [^3^H]2-deoxyglucose. Glut-4: Glucose transporter-4. siRNA: small interfering RNA. WM: wartmanin.

## Competing interests

The authors declare that they have no competing interests.

## Authors' contributions

BB designed and executed experiments, analysed data and drafted the manuscript. CSD conceived the study, designed, coordinated experiments, written and finalized the manuscript. All authors read and approved the final manuscript.

## Supplementary Material

Additional File 1 **C2C12 cells expressing GFP-Glut-4**. C2C12 cells were transfected with GFP-Glut-4 plasmid and expression of Glut-4 (GFP: Green), Actin (Phalloidin Texas Red: Red) and Nucleus (DAPI: Blue) were examined in proliferated and differentiated cells. No green fluorescence was observed in untransfected, proliferated C2C12 wild type cells **(A) **whereas transfected cells, expressing GFP-Glut-4 (C2Glut-4^wt/+^), emitted green fluorescence in **(B) **proliferated C2Glut-4^wt/+ ^and **(C) **Differentiated C2Glut-4^wt/+ ^cells. Intact nucleus as observed in DAPI stained transfected cells confirmed no cell death due to transfection. Images are representative of multiple fields of 3 independent experiments. Bar corresponds to 10 μm. Click here for file

Additional File 2 **Insulin-mediated translocation of GFP-Glut-4 molecules in C2Glut-4^wt/+ ^cells**. C2Glut-4^wt/+ ^cells were differentiated and stimulated with and without insulin (100 nM) for 30 min and translocation of Glut-4 were examined. In absence of insulin Glut-4 molecules remained concentrated in cytoplasm near the nucleus **(A) **whereas they dispersed in the cytoplasm in the process of translocation to the membrane after insulin stimulation **(B)**. Blue arrow head indicates Glut-4 molecules; Red arrow head indicates the peripheral region without Glut-4 molecules. Images are representative of multiple fields of 3 independent experiments. Bar is 5 μm, 10 μm for inset. Click here for file

Additional File 3 **Transfection of FAK wild type plasmid and siRNA for FAK in C2Glut-4^wt/+ ^cells**. C2Glut-4^wt/+ ^cells were transfected with FAK wild type plasmid and FAK specific siRNA (400 nM). **(A) **Cells were differentiated, lysed and western immunoblotted with anti-FAK and anti-Glut-4 antibodies. Blots were stripped and re-probed with anti-actin antibody. Lane 1: C2C12 wild type myotubes, Lane 2: C2Glut-4^wt/+ ^cells, Lane 3: C2Glut-4^wt/+^FAK^wt/+ ^and Lane 4: C2Glut-4^wt/+^FAK^wt/-^. **(B) **Morphological examination of differentiated transfected and untransfected cells. No change in cellular morphology of transfected and untransfected cells was observed. Images are representative of multiple fileds of 3 experiments. Bar is 20 μm. Click here for file

Additional File 4 **Effect of insulin on actin remodeling and its colocalization with Glut-4 in C2Glut-4^wt/+ ^cells**. C2Glut-4^wt/+ ^cells were differentiated under MF and MFI condition, serum starved (4 h) and stimulated with and without 100 nM insulin for 30 min at 37°C followed by fixation and permeabilization. Actin filaments, nucleus, Glut-4 were labeled with Phalloidin Texas Red, DAPI and GFP respectively. Images were captured from different fields and a representative image of 3 experiments is presented. Bar is 5 μm. **(A) **C2Glut-4^wt/+ ^cells differentiated under MF condition (represent Fig. 2A); **(B) **C2Glut-4^wt/+ ^cells differentiated under MF condition stimulated with insulin (represent Fig. 2D); **(C) **C2Glut-4^wt/+ ^cells differentiated under MFI condition (represent Fig. 2G); **(D) **C2Glut-4^wt/+ ^cells differentiated under MFI condition stimulated with insulin (represent Fig. 2J). Yellow arrow head represents Glut-4 molecules, White arrow head represent actin filaments and Blue arrow head indicates colocalized actin with Glut-4. Click here for file

Additional File 5 **Effect of FAK overexpression on actin remodeling and its colocalization with Glut-4, after insulin stimulation**. Cells overexpressing FAK (C2Glut-4^wt/+^FAK^wt/+^) were differentiated under MF and MFI condition, serum starved (4 h) and stimulated with and without 100 nM insulin for 30 min at 37°C followed by fixation and permeabilization. Actin filaments, nucleus, Glut-4 were labeled with Phalloidin Texas Red, DAPI and GFP, respectively. Images were captured from different fields and a representative image of 3 experiments is presented. Bar is 5 μm. **(A) **C2Glut-4^wt/+^FAK^wt/+ ^cells differentiated under MF condition (represent Fig. 2B); **(B) **C2Glut-4^wt/+^FAK^wt/+ ^cells differentiated under MF condition stimulated with insulin (represent Fig. 2E); **(C) **C2Glut-4^wt/+^FAK^wt/+ ^differentiated under MFI condition (represent Fig. 2H); **(D) **C2Glut-4^wt/+^FAK^wt/+ ^cells differentiated under MFI condition stimulated with insulin (represent Fig. 2K). Yellow arrow head represents Glut-4 molecules, White arrow head represent actin filaments and Blue arrow head indicates colocalized actin with Glut-4. Click here for file

Additional File 6 **Effect of FAK silencing on insulin-mediated actin remodeling and Glut-4 translocation**. FAK silenced cells (C2Glut-4^wt/+^FAK^wt/-^) were differentiated under MF and MFI condition, serum starved (4 h) and stimulated with and without 100 nM insulin for 30 min at 37°C followed by fixation and permeabilization. Actin filaments, nucleus, Glut-4 were labeled with Phalloidin Texas Red, DAPI and GFP, respectively. Images were captured from different fields and a representative image of 3 experiments was presented. Bar is 5 μm. **(A) **C2Glut-4^wt/+^FAK^wt/- ^cells differentiated under MF condition (represent Fig. 2C); **(B) **C2Glut-4^wt/+^FAK^wt/- ^cells differentiated under MF condition stimulated with insulin (represent Fig. 2F); **(C) **C2Glut-4^wt/+^FAK^wt/- ^differentiated under MFI condition (represent Fig. 2I); **(D) **C2Glut-4^wt/+^FAK^wt/- ^cells differentiated under MFI condition stimulated with insulin (represent Fig. 2L). Yellow arrow head represents Glut-4 molecules, White arrow head represent actin filaments and Blue arrow head indicates colocalized actin with Glut-4. Click here for file

Additional File 7 **Possible signal pathway for FAK-regulated insulin signaling.** Click here for file
