# Supplementary figures and images for: Focal Adhesion Kinase contributes to insulin-induced actin reorganization into a mesh harboring Glucose transporter-4 in insulin resistant skeletal muscle cells
Source: BMC Cell Biol. 2008 Sep 4;9:48. doi: 10.1186/1471-2121-9-48 (PMC2551595; doi:10.1186/1471-2121-9-48)

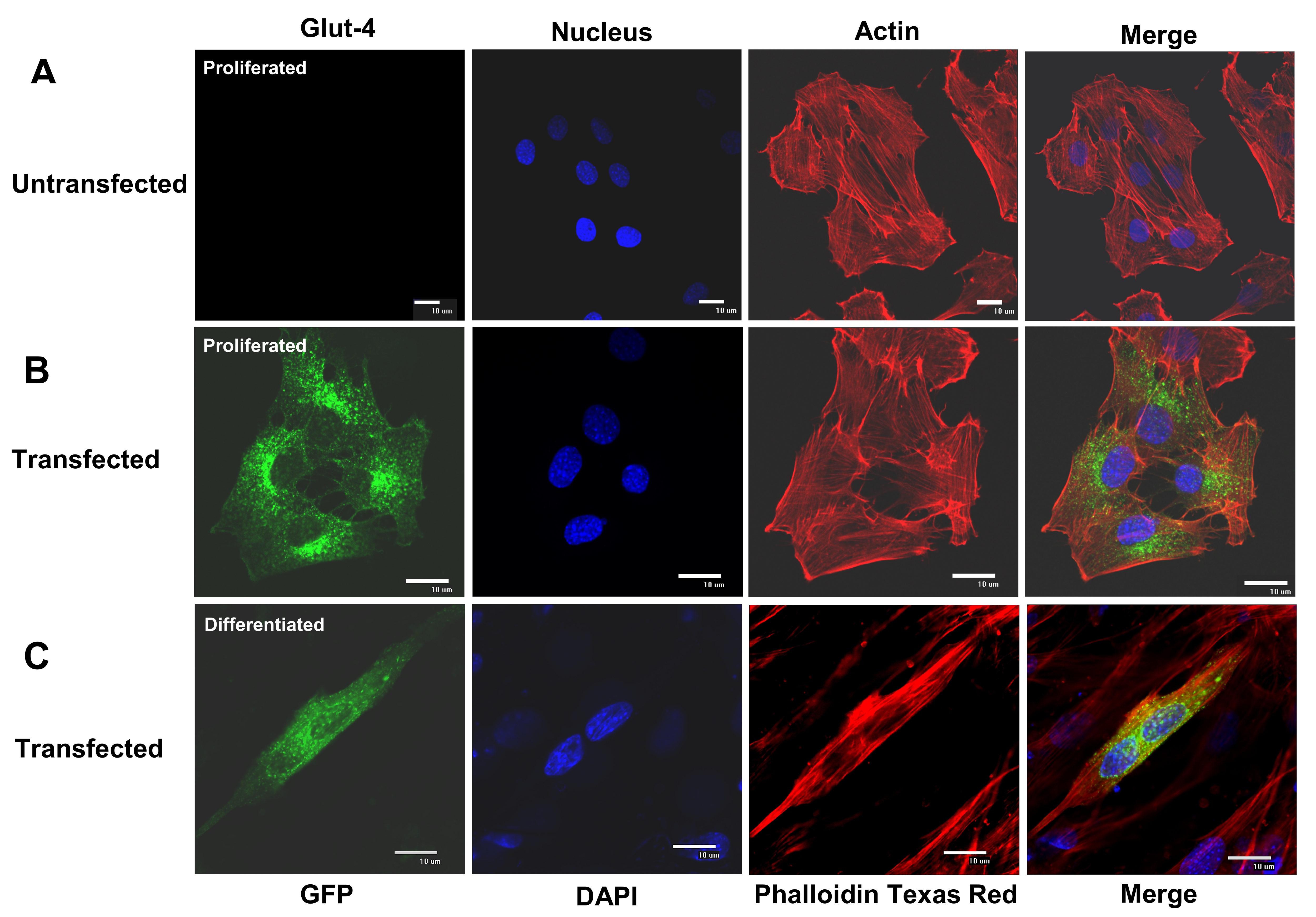

Supplement: Additional File 1 — C2C12 cells expressing GFP-Glut-4. C2C12 cells were transfected with GFP-Glut-4 plasmid and expression of Glut-4 (GFP: Green), Actin (Phalloidin Texas Red: Red) and Nucleus (DAPI: Blue) were examined in proliferated and differentiated cells. No green fluorescence was observed in untransfected, proliferated C2C12 wild type cells (A) whereas transfected cells, expressing GFP-Glut-4 (C2Glut-4wt/+), emitted green fluorescence in (B) proliferated C2Glut-4wt/+ and (C) Differentiated C2Glut-4wt/+ cells. Intact nucleus as observed in DAPI stained transfected cells confirmed no cell death due to transfection. Images are representative of multiple fields of 3 independent experiments. Bar corresponds to 10 μm. [file 1471-2121-9-48-S1.jpeg]

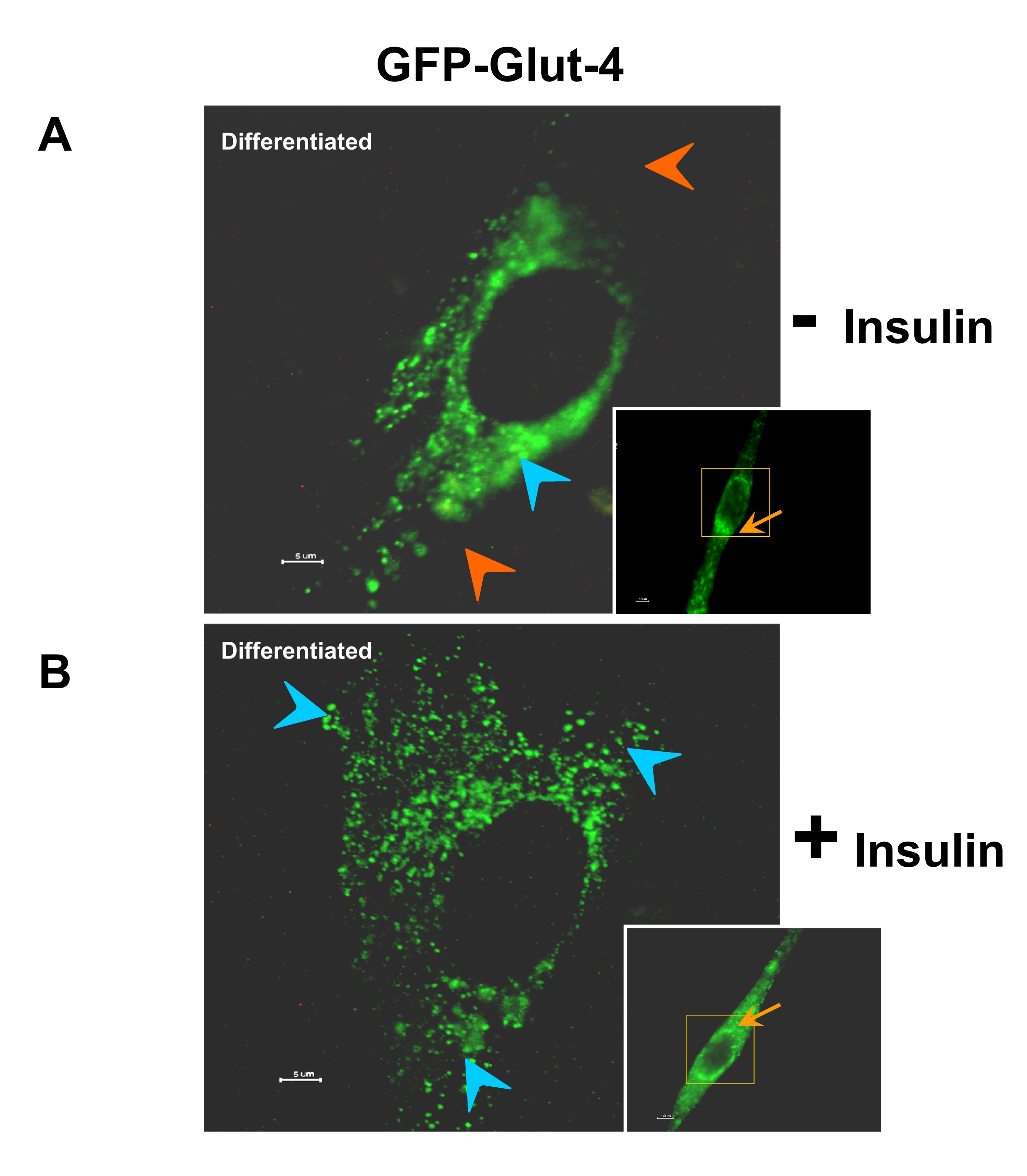

Supplement: Additional File 2 — Insulin-mediated translocation of GFP-Glut-4 molecules in C2Glut-4wt/+ cells. C2Glut-4wt/+ cells were differentiated and stimulated with and without insulin (100 nM) for 30 min and translocation of Glut-4 were examined. In absence of insulin Glut-4 molecules remained concentrated in cytoplasm near the nucleus (A) whereas they dispersed in the cytoplasm in the process of translocation to the membrane after insulin stimulation (B). Blue arrow head indicates Glut-4 molecules; Red arrow head indicates the peripheral region without Glut-4 molecules. Images are representative of multiple fields of 3 independent experiments. Bar is 5 μm, 10 μm for inset. [file 1471-2121-9-48-S2.jpeg]

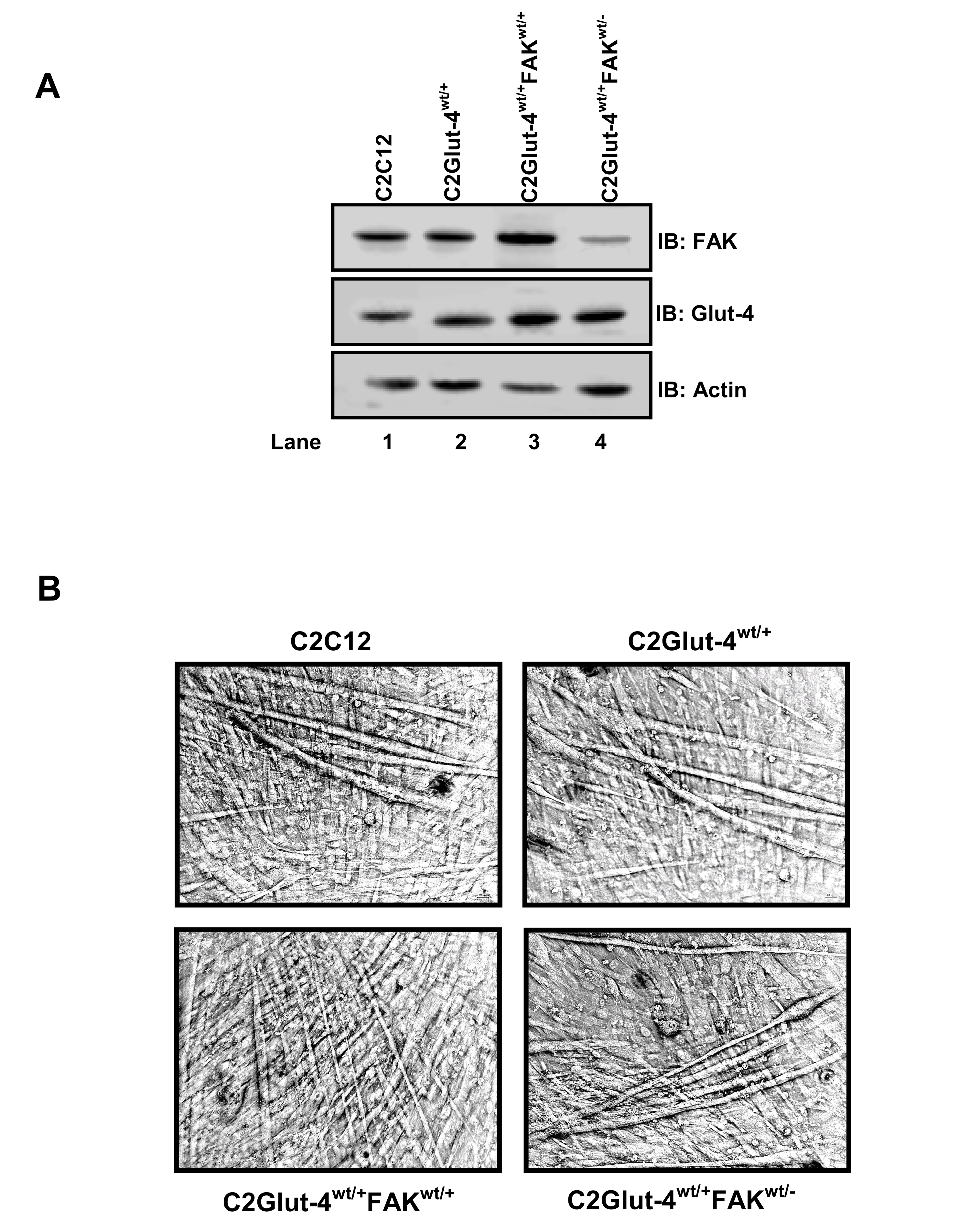

Supplement: Additional File 3 — Transfection of FAK wild type plasmid and siRNA for FAK in C2Glut-4wt/+ cells. C2Glut-4wt/+ cells were transfected with FAK wild type plasmid and FAK specific siRNA (400 nM). (A) Cells were differentiated, lysed and western immunoblotted with anti-FAK and anti-Glut-4 antibodies. Blots were stripped and re-probed with anti-actin antibody. Lane 1: C2C12 wild type myotubes, Lane 2: C2Glut-4wt/+ cells, Lane 3: C2Glut-4wt/+FAKwt/+ and Lane 4: C2Glut-4wt/+FAKwt/-. (B) Morphological examination of differentiated transfected and untransfected cells. No change in cellular morphology of transfected and untransfected cells was observed. Images are representative of multiple fileds of 3 experiments. Bar is 20 μm. [file 1471-2121-9-48-S3.jpeg]

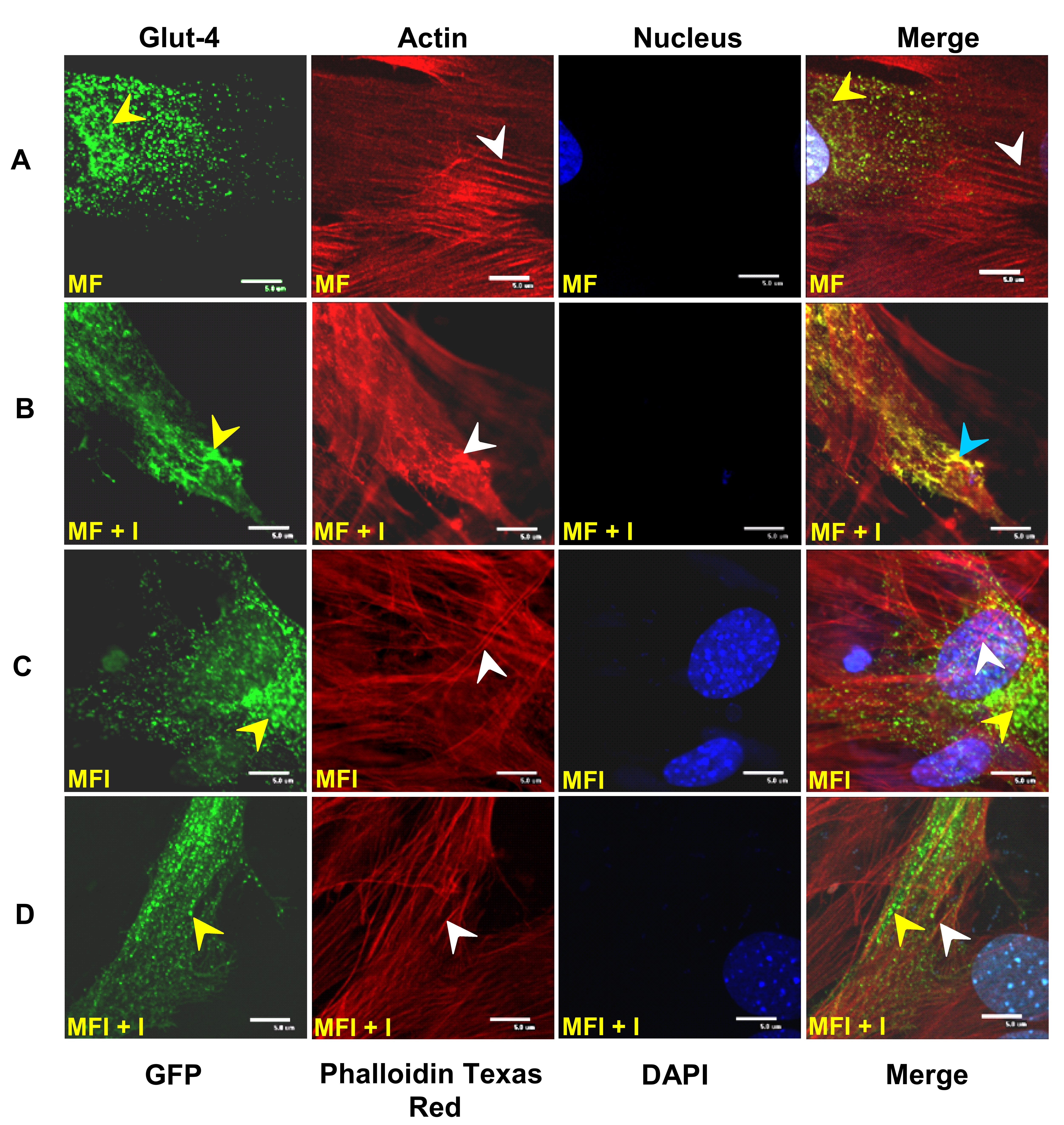

Supplement: Additional File 4 — Effect of insulin on actin remodeling and its colocalization with Glut-4 in C2Glut-4wt/+ cells. C2Glut-4wt/+ cells were differentiated under MF and MFI condition, serum starved (4 h) and stimulated with and without 100 nM insulin for 30 min at 37°C followed by fixation and permeabilization. Actin filaments, nucleus, Glut-4 were labeled with Phalloidin Texas Red, DAPI and GFP respectively. Images were captured from different fields and a representative image of 3 experiments is presented. Bar is 5 μm. (A) C2Glut-4wt/+ cells differentiated under MF condition (represent Fig. 2A); (B) C2Glut-4wt/+ cells differentiated under MF condition stimulated with insulin (represent Fig. 2D); (C) C2Glut-4wt/+ cells differentiated under MFI condition (represent Fig. 2G); (D) C2Glut-4wt/+ cells differentiated under MFI condition stimulated with insulin (represent Fig. 2J). Yellow arrow head represents Glut-4 molecules, White arrow head represent actin filaments and Blue arrow head indicates colocalized actin with Glut-4. [file 1471-2121-9-48-S4.jpeg]

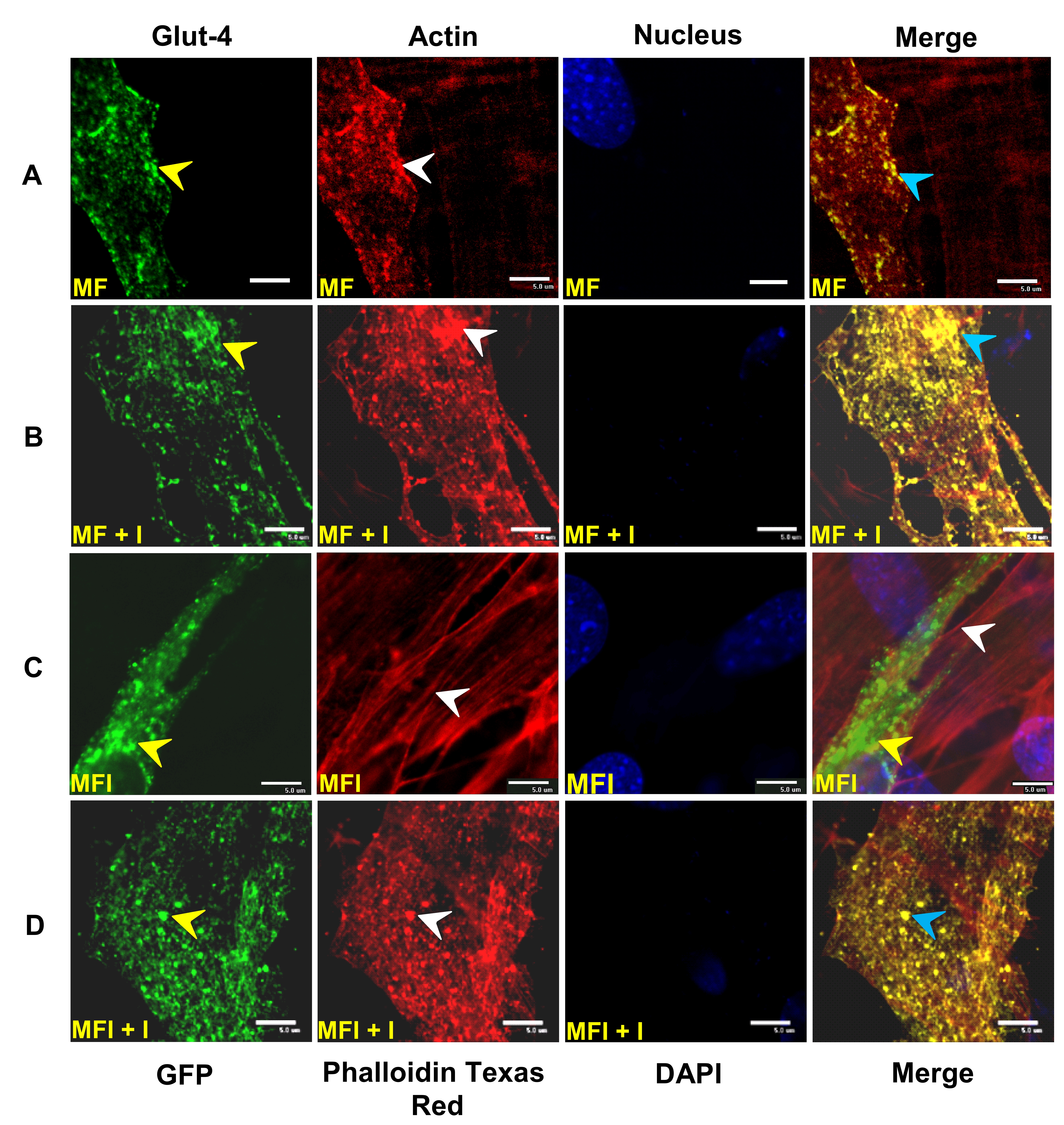

Supplement: Additional File 5 — Effect of FAK overexpression on actin remodeling and its colocalization with Glut-4, after insulin stimulation. Cells overexpressing FAK (C2Glut-4wt/+FAKwt/+) were differentiated under MF and MFI condition, serum starved (4 h) and stimulated with and without 100 nM insulin for 30 min at 37°C followed by fixation and permeabilization. Actin filaments, nucleus, Glut-4 were labeled with Phalloidin Texas Red, DAPI and GFP, respectively. Images were captured from different fields and a representative image of 3 experiments is presented. Bar is 5 μm. (A) C2Glut-4wt/+FAKwt/+ cells differentiated under MF condition (represent Fig. 2B); (B) C2Glut-4wt/+FAKwt/+ cells differentiated under MF condition stimulated with insulin (represent Fig. 2E); (C) C2Glut-4wt/+FAKwt/+ differentiated under MFI condition (represent Fig. 2H); (D) C2Glut-4wt/+FAKwt/+ cells differentiated under MFI condition stimulated with insulin (represent Fig. 2K). Yellow arrow head represents Glut-4 molecules, White arrow head represent actin filaments and Blue arrow head indicates colocalized actin with Glut-4. [file 1471-2121-9-48-S5.jpeg]

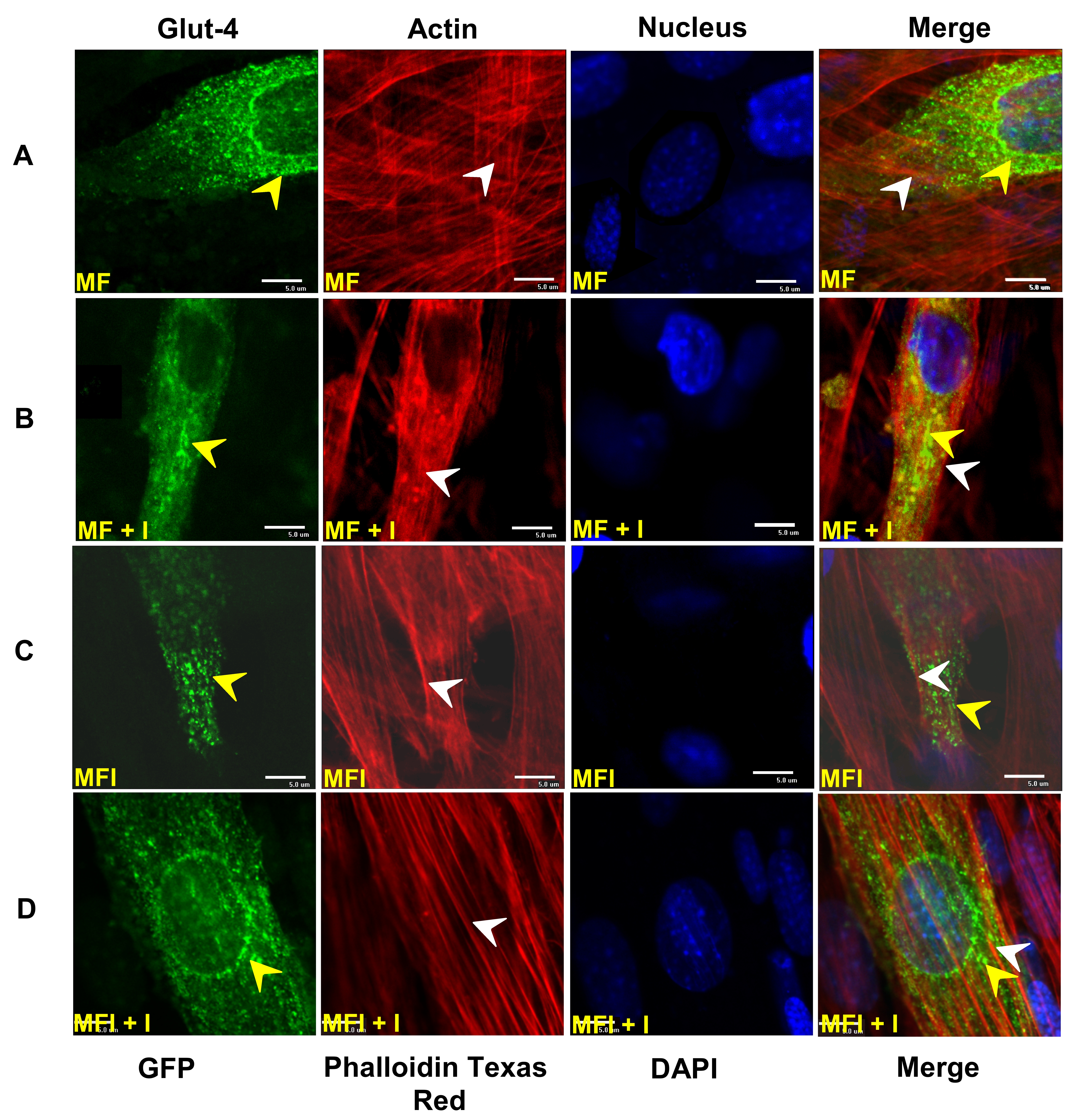

Supplement: Additional File 6 — Effect of FAK silencing on insulin-mediated actin remodeling and Glut-4 translocation. FAK silenced cells (C2Glut-4wt/+FAKwt/-) were differentiated under MF and MFI condition, serum starved (4 h) and stimulated with and without 100 nM insulin for 30 min at 37°C followed by fixation and permeabilization. Actin filaments, nucleus, Glut-4 were labeled with Phalloidin Texas Red, DAPI and GFP, respectively. Images were captured from different fields and a representative image of 3 experiments was presented. Bar is 5 μm. (A) C2Glut-4wt/+FAKwt/- cells differentiated under MF condition (represent Fig. 2C); (B) C2Glut-4wt/+FAKwt/- cells differentiated under MF condition stimulated with insulin (represent Fig. 2F); (C) C2Glut-4wt/+FAKwt/- differentiated under MFI condition (represent Fig. 2I); (D) C2Glut-4wt/+FAKwt/- cells differentiated under MFI condition stimulated with insulin (represent Fig. 2L). Yellow arrow head represents Glut-4 molecules, White arrow head represent actin filaments and Blue arrow head indicates colocalized actin with Glut-4. [file 1471-2121-9-48-S6.jpeg]

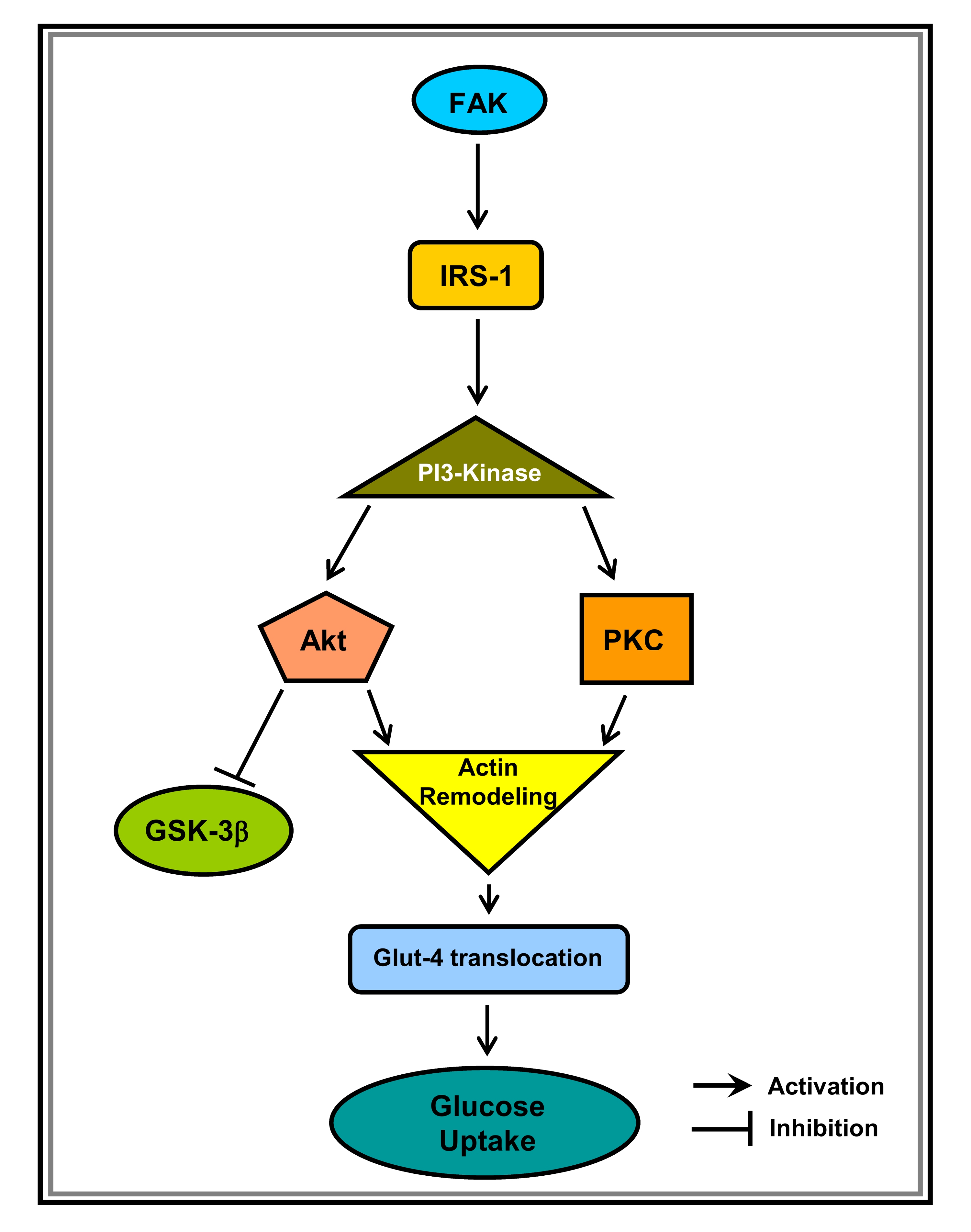

Supplement: Additional File 7 — Possible signal pathway for FAK-regulated insulin signaling. [file 1471-2121-9-48-S7.jpeg]
